# Peak expiratory flow as an endpoint for clinical trials in asthma: a comparison with FEV_1_

**DOI:** 10.1186/s12931-019-1119-6

**Published:** 2019-07-18

**Authors:** David M. G. Halpin, Eli O. Meltzer, Wendelgard Pisternick-Ruf, Petra Moroni-Zentgraf, Michael Engel, Liliana Zaremba-Pechmann, Thomas Casale, J. Mark FitzGerald

**Affiliations:** 1 University of Exeter Medical School, College of Medicine and Health, University of Exeter, Exeter, EX1 2LU UK; 2 Allergy and Asthma Medical Group and Research Center, University of California, San Diego, CA USA; 3 Boehringer Ingelheim Pharma GmbH & Co. KG, Biberach an der Riss, Germany; 4 Boehringer Ingelheim Pty Ltd, Sydney, Australia; 5 Boehringer Ingelheim International GmbH, Ingelheim am Rhein, Germany; 6 Division of Allergy and Immunology, University of South Florida Morsani College of Medicine, Tampa, FL USA; 7 Centre for Heart and Lung Health, Vancouver, Canada

**Keywords:** Asthma, Correlation, FEV_1_, Home-measurement, In-clinic measurement, PEF, Tiotropium

## Abstract

**Background:**

The primary lung function endpoint in clinical trials in adolescent and adult patients with asthma is usually forced expiratory volume in one second (FEV_1_). The objective of our analysis was to assess whether peak expiratory flow (PEF) is a suitable alternative primary lung function endpoint.

**Methods:**

For this assessment, we calculated post hoc the correlation between pre-dose FEV_1_ and pre-dose PEF measured under supervision in the clinic and, for both lung function parameters, the correlations between supervised clinic and unsupervised home measurements, using the results from the 8 Phase III parallel-group trials of the global clinical development programme with tiotropium Respimat® in patients with asthma aged 12 to 75 years.

**Results:**

Across all 8 trials included in this analysis, changes in lung function from baseline correlated well between pre-dose FEV_1_ and pre-dose PEF when both were measured under supervision in the clinic. Correlation between supervised in-clinic and unsupervised home measurements was stronger for pre-dose PEF than for pre-dose FEV_1_.

**Conclusions:**

Pre-dose PEF measured at home could be an alternative primary lung function endpoint for trials in adolescent and adult patients with asthma. Using home-measured PEF could facilitate trial conduct and improve the convenience for patients by relocating scheduled assessments from the clinic to the patient’s home.

**Trial registration:**

Adolescents aged 12 to 17 years: RubaTinA-asthma® (NCT01257230), PensieTinA-asthma® (NCT01277523).

Adults aged 18 to 75 years: GraziaTinA-asthma® (NCT01316380), MezzoTinA-asthma® (NCT01172808/NCT01172821), CadenTinA-asthma® (NCT01340209), PrimoTinA-asthma® (NCT00772538/NCT00776984).

All from Clinicaltrials.gov (https://clinicaltrials.gov/).

**Electronic supplementary material:**

The online version of this article (10.1186/s12931-019-1119-6) contains supplementary material, which is available to authorized users.

## Background

Spirometry is one of the fundamental outcome measures used in asthma studies. It provides an objective and highly reproducible measure of airflow limitation caused by smooth muscle contraction or structural changes [1]. Forced expiratory volume in 1 s (FEV_1_) is recommended as the primary endpoint for studies of bronchodilator therapy by the American Thoracic Society (ATS) and the European Respiratory Society (ERS) in their official statement on asthma control and exacerbations [1]. Pre-bronchodilator FEV_1_, i.e. the FEV_1_ recorded after withholding bronchodilators for their duration of action, is a strong, independent predictor of future exacerbation risk, and has been used in the majority of asthma clinical trials as the primary lung function endpoint in recent decades [1]. This is in line with regulatory recommendations for clinical trials in asthma that also consider pre-bronchodilator FEV_1_ as the most suitable variable [2].

Peak expiratory flow (PEF) is also an accepted spirometric measure that provides information about the level of airflow obstruction, both initially and in clinical trials to monitor asthma control and treatment responses [3]; however, it is generally considered more appropriate for home monitoring of lung function [2].

Both FEV_1_ and PEF can be measured under supervision in the clinic or unsupervised at home. In clinical trials, home measurements could increase the convenience and reduce the time and logistical burden for trial participants. So far, home-measured FEV_1_ or PEF have mainly been used in studies to provide complementary information to symptom diaries or clinic FEV_1_ [1].

The main objective of our analysis was to assess whether PEF, measured either at home or in the clinic, could be used as an alternative lung function endpoint in asthma clinical trials. In addition, the suitability of home-measured FEV_1_ as a lung function endpoint was investigated. For this assessment, we calculated post hoc the correlation between pre-dose FEV_1_ and pre-dose PEF measured under supervision in the clinic and, for both lung function parameters, the correlations between supervised clinic and unsupervised home measurements, using the results from the 8 Phase III parallel-group trials of the global clinical development programme with tiotropium Respimat® in patients with asthma aged 12–75 years. Tiotropium Respimat® has demonstrated improvements in lung function, asthma exacerbation risk and asthma control, is approved in the European Union [4] and in the United States [5], and is indicated as an add-on maintenance bronchodilator treatment in patients aged 6 years and older with severe asthma who experienced one or more severe asthma exacerbations in the preceding year.

## Methods

### Trial design and trial population

This exploratory post hoc analysis included lung function data from all Phase III parallel-group trials of the global Boehringer Ingelheim programme of tiotropium Respimat® in asthma in patients aged 12 years and older [6–11]. These were 8 randomised, double-blind, placebo-controlled trials of between 12 and 52 weeks’ duration. All trials included once-daily tiotropium Respimat® 5 μg and placebo, 6 trials also included once-daily tiotropium Respimat® 2.5 μg, and 2 trials included twice-daily salmeterol as a fourth treatment arm. All trial medication was administered as add-on to ICS, with or without other controller medications such as long-acting β_2_-agonists (LABAs) or leukotriene receptor antagonists (LTRAs). Out of a total of 4550 treated patients aged 12 to 75 years with symptomatic persistent asthma of different severities, 4525 patients had baseline and at least 1 on-treatment efficacy measurement, and were evaluated for efficacy. Further details on the trial design, the required minimum maintenance therapy and the treatment groups are summarised in Table 1.

**Table 1:** Overview of placebo-controlled, parallel-group Phase III trials with tiotropium Respimat® in patients aged 12 to 75 years with persistent asthma

Trial	Severity of persistent asthma	Daily required minimum maintenance therapy	ClinicalTrials.gov identifier; main publication	Treatment duration [weeks]	Tiotropium Respimat®		Sal 50	Time of once-daily tiotropium dosing	Number of patients evaluated for efficacy (treated)
					5 μg	2.5 μg			
*Adolescents (12 to 17 years)*									
RubaTinA-asthma®	Moderate	400–800 μg BUD-eq^a,b^	NCT01257230; Hamelmann et al. 2016 [6]	48	✓	✓	–	Evening	397 (397)
PensieTinA-asthma®	Severe	400–800 μg BUD-eq^b^ + 2 additional controllers^c^/ 800–1600 μg BUD-eq^d^ + 1 additional controller^e,f^	NCT01277523; Hamelmann et al., 2016 [7]	12	✓	✓	–	Evening	392 (392)
*Adults (18 to 75 years)*									
GraziaTinA-asthma**®**	Mild	200–400 μg BUD-eq	NCT01316380; Paggiaro et al., 2016 [8]	12	✓	✓	–	Evening	464 (464)
MezzoTinA-asthma® (2 replicate trials)	Moderate	400–800 BUD-eq^a^	NCT01172808/ NCT01172821; Kerstjens et al., 2015 [9]	24	✓	✓	✓	Evening	2081 (2100)
CadenTinA-asthma®	Moderate to severe	400–800 μg BUD-eq^a,g^	NCT01340209; Ohta et al., 2015 [10]	52	✓	✓	–	Evening	284 (285)
PrimoTinA-asthma**®** (2 replicate trials)	Severe	≥800 μg BUD-eq + LABA^a,f^	NCT00772538/ NCT00776984; Kerstjens et al., 2012 [11]	48	✓	–	–	Morning	907 (912)
								Total	4525 (4550)

^a^LTRA permitted. ^b^For 12–14-year-olds, 200–400 μg BUD-eq was also considered an appropriate medium dose of inhaled corticosteroid.^c^Two controllers of either a LABA, an LTRA or a sustained-release theophylline required. ^d^For 12–14-year-olds, > 400 μg BUD-eq was also considered an appropriate high dose of inhaled corticosteroid. ^e^LABA or LTRA required. ^f^Sustained-release theophylline permitted. ^g^LABA permitted

BUD-eq = budesonide or equivalent dose; LABA = long-acting β_2_-agonist; LTRA = leukotriene receptor agonist; Sal 50 = salmeterol 50 μg twice daily (morning and evening dosing)

### Lung function assessments

Supervised measurements of FEV_1_ and PEF at clinic visits were performed for all trials, except PrimoTinA-asthma®, using MasterScope® computed tomography spirometers (eResearch Technology [ERT]). For PrimoTinA-asthma®, only FEV_1_ was measured under supervision, using the sites’ own equipment; PEF was not measured under supervision. For all trials, spirometers and their use, including daily calibration, had to meet the ATS/ERS criteria [12]. Pulmonary function tests were to be performed at approximately the same time of the day before administration of maintenance ICS therapy and trial medication.

Unsupervised measurements of FEV_1_ and PEF at home were performed using an electronic peak flow meter (Asthma Monitor® [ERT]). All patients were trained in the use of the device at the screening and randomisation visits in the clinic. For all trials, pulmonary function tests were to be performed at approximately the same time of the day, prior to administration of maintenance ICS therapy and trial medication.

For both supervised and unsupervised measurements, the highest FEV_1_ and PEF values out of 3 acceptable manoeuvres (not necessarily from the same manoeuvre) were used for the evaluation.

### Correlation analyses

We analysed the correlations between pre-dose FEV_1_ and pre-dose PEF measured under supervision in the clinic and between supervised in-clinic and unsupervised home measurements for both PEF and FEV_1_. For the correlation analyses, data of the two pairs of replicate trials in adults with moderate (MezzoTinA-asthma®) or severe (PrimoTinA-asthma®) asthma were pooled; the other analyses were performed by trial. Since in PrimoTinA-asthma® no in-clinic measurement of pre-dose PEF was performed, correlation analyses in these pooled trials were limited to pre-dose FEV_1_.

For the calculation of the correlation coefficients, the response values (i.e. the change from baseline of all treatment groups of the respective trial) were considered. As tiotropium is a long-acting bronchodilator with once-daily dosing, the lung function parameters measured at the end of the dosing interval are relevant to support efficacy. Therefore, the pre-dose values were used for the correlation assessment, although, in most of the trials, both FEV_1_ peak and trough were included as primary and key secondary lung function endpoints. For the in-clinic measured values, the pre-dose values included in the calculation were those assessed just prior to the next dose. For the home-measured values, the weekly means of the values assessed daily prior to dosing were used, i.e. morning FEV_1_ and morning PEF for the trials with morning dosing, and evening FEV_1_ and evening PEF for the trials with evening dosing.

**Fig. 1:**
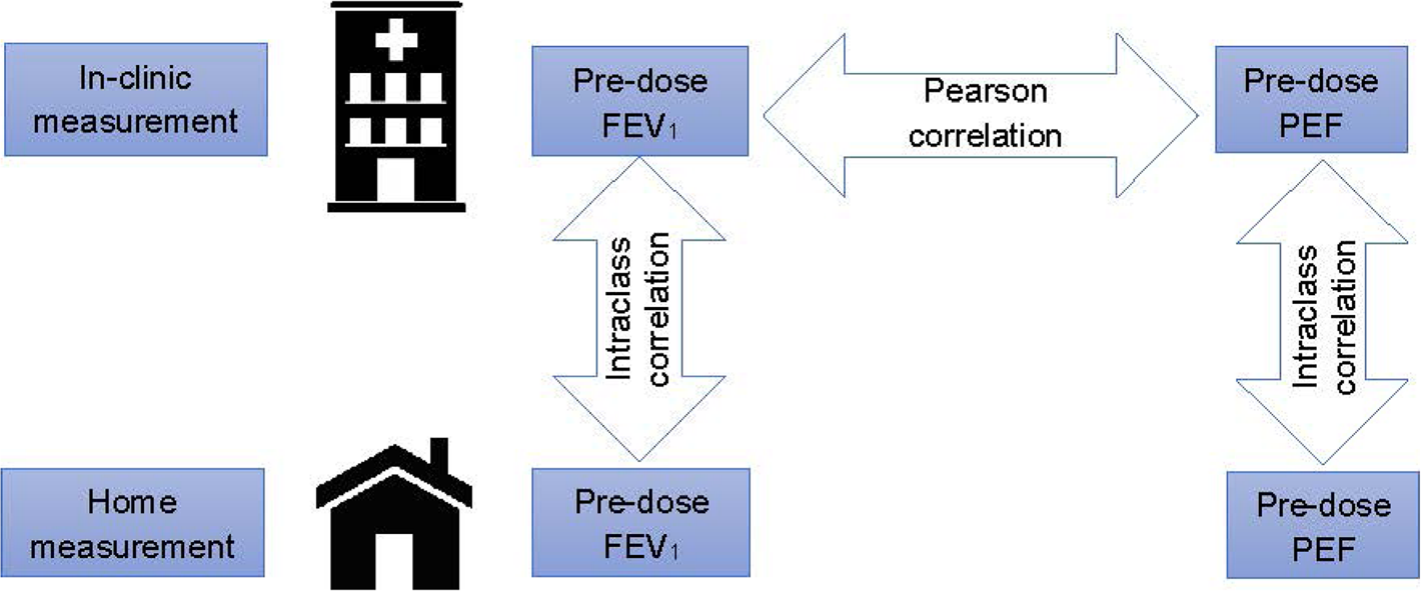
Overview of correlation analyses. FEV_1_ = forced expiratory volume in one second; PEF = peak expiratory flow

The statistical measures used for the correlation analyses are summarised in Fig. 1. For the two different lung function parameters, pre-dose FEV_1_ and pre-dose PEF, the Pearson correlation (PCC) was calculated [13]. For the different assessments (in-clinic vs. home) of the same variable (either pre-dose FEV_1_ or pre-dose PEF), the intraclass correlation (ICC) was calculated [13].

To assess the extent of correlation, the correlation coefficients (PCC or ICC) were interpreted as follows: > 0.9 to 1.0 very high, > 0.7 to 0.9 high, and > 0.5 to 0.7 moderate correlation.

## Results

Across the trials, patients had a broad range of asthma severities. Key baseline demographics and disease characteristics are summarised by trial in Table 2. Within each trial, baseline demographics and disease characteristics were comparable between the treatment groups (see published manuscripts [6–11]). Most of the adolescent or adult patients were White or Asian and had never smoked. Mean duration of asthma was about 8 years in the trials in adolescents and from around 16 years in adult patients with mild persistent asthma to around 30 years in adult patients with severe persistent asthma. Lung function in terms of FEV_1_ and PEF at baseline was in line with the different ranges of asthma severity. In summary, the patients were representative of adolescent and adult patients with different severities of persistent asthma in the real-world setting.

**Table 2:** Summary of baseline demographics and disease characteristics in Phase III trials with tiotropium Respimat® in patients aged 12 to 75 years with persistent asthma – all treated patients

Trial	Age	Sex	Race	Never smoked	Duration of asthma	FEV_1_ at baseline	PEF^d^ at baseline
*(Treated patients)*	*Mean ± SD [years]*	*Male, n (%); Female, n (%)*	*White, n (%); Asian, n (%); Black* ^*a*^ *, n (%); American Indian* ^*b*^ *, n (%); Hawaiian* ^*c*^ *, n (%)*	*n (%)*	*Mean ± SD [years]*	*Mean ± SD [L]/[% pred.]*	*Weekly mean ± SD [mL/min]*
*Adolescents (12 to 17 years)*							
RubaTinA-asthma® (*N* = 397)	14.3 ± 1.7	258 (65.0); 139 (35.0)	368 (92.7); 13 (3.3); 14 (3.5); 2 (0.5); 0	396 (99.7)	7.9 ± 4.1	2.75 ± 0.66/82.8 ± 10.6	360.0 ± 91.1
PensieTinA-asthma® (*N* = 392)	14.2 ± 1.7	242 (61.7); 150 (38.3)	371 (94.6); 10 (2.6); 8 (2.0); 3 (0.8); 0	392 (100)	7.8 ± 3.9	2.53 ± 0.62/79.5 ± 11.5	346.1 ± 91.8
*Adults (18 to 75 years)*							
GraziaTinA-asthma® (*N* = 464)	42.9 ± 13.0	183 (39.4); 281 (60.6)	362 (78.0); 85 (18.3); 1 (0.2); 16 (3.4); 0	382 (82.3)	16.2 ± 11.9	2.42 ± 0.71/77.7 ± 11.9	369.8 ± 114.9
MezzoTinA-asthma® (*N* = 2100)	43.1 ± 12.9	861 (41.0); 1239 (59.0)	1005 (47.9); 893 (42.5); 81 (3.9); 118 (5.6); 3 (0.1)	1756 (83.6)	21.8 ± 14.3	2.27 ± 0.65/75.1 ± 11.5	349.6 ± 117.2
CadenTinA-asthma® (*N* = 285)	44.5 ± 12.7	109 (38.2); 176 (61.8)	0; 285 (100.0); 0; 0; 0	214 (75.1)	22.4 ± 14.2	2.28 ± 0.65/80.2 ± 12.5	375.5 ± 118.4
PrimoTinA-asthma® (*N* = 912)	53.0 ± 12.4	361 (39.6); 551 (60.4)	759 (83.2); 103 (11.3); 47 (5.2); 1 (0.1); 2 (0.2)	692 (75.9)	30.3 ± 13.9	1.60 ± 0.54/56.0 ± 13.1	270.7 ± 111.1

^a^Or African American. ^b^Or Alaska Native. ^c^Or Pacific Islander. ^d^Measured with AM® device at baseline at the same time of day before dosing occurred, i.e. morning PEF in the trials with morning dosing (PrimoTinA-asthma®), evening PEF in the trials with evening dosing (RubaTinA-asthma®, PensieTinA-asthma®, GraziaTinA-asthma®, MezzoTinA-asthma®, CadenTinA-asthma®)

FEV_1_ = forced expiratory volume in one second; PEF = peak expiratory flow; SD = standard deviation

**Fig. 2:**
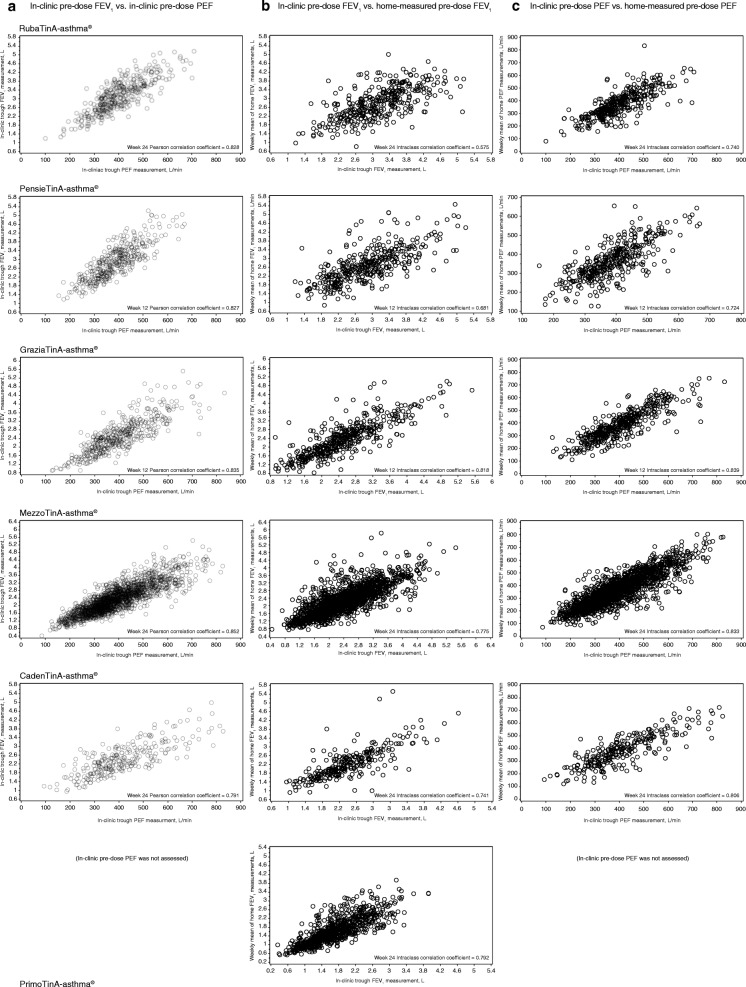
Correlation analysis results between pre-dose FEV_1_ and pre-dose PEF, measured either under supervision in the clinic or unsupervised at home, at the time of primary efficacy evaluation. Phase III trials with tiotropium Respimat® in patients aged 12–75 years with persistent asthma – all patients analysed for efficacy. (**a**) In-clinic pre-dose FEV_1_ vs. in-clinic pre-dose PEF; (**b**) in-clinic pre-dose FEV_1_ vs. home-measured pre-dose FEV_1_^a^; (**c**) in-clinic pre-dose PEF vs. home-measured pre-dose PEF^b^. ^a^Home-measured pre-dose FEV_1_: weekly mean morning FEV_1_ in the trials with morning dosing (PrimoTinA-asthma®) and weekly mean evening FEV_1_ in the trials with evening dosing (RubaTinA-asthma®, PensieTinA-asthma®, GraziaTinA-asthma®, MezzoTinA-asthma®, CadenTinA-asthma®), measured with AM device. ^b^Home-measured pre-dose PEF: weekly mean morning PEF in the trials with morning dosing (PrimoTinA-asthma®) and weekly mean evening PEF in the trials with evening dosing (RubaTinA-asthma®, PensieTinA-asthma®, GraziaTinA-asthma®, MezzoTinA-asthma®, CadenTinA-asthma®), measured with AM device. PEF was not measured at clinic visits in the PrimoTinA-asthma® study. FEV_1_ = forced expiratory volume in one second; PEF = peak expiratory flow

Correlation between pre-dose FEV_1_ and pre-dose PEF, when both parameters were measured under supervision in the clinic, was consistently high for all trials: the PCCs ranged from 0.773 to 0.852 across all trials at all time points; see Table 3 and Additional file 1: Table S1 for details. The scatter plots in Fig. 2 visualise the strong correlation between supervised pre-dose FEV_1_ and supervised pre-dose PEF at the time of primary efficacy evaluation across all trials; for CadenTinA-asthma®, which did not have a primary efficacy evaluation, Week 24 was used for the analysis.

**Table 3:** Correlation analysis results (range over different time points) from Phase III trials with tiotropium Respimat® in patients aged 12–75 years with persistent asthma – all patients evaluated for efficacy

Trial	In-clinic pre-dose FEV_1_ vs. in-clinic pre-dose PEF	In-clinic pre-dose FEV_1_ vs. home-measured pre-dose FEV_1_	In-clinic pre-dose PEF vs. home-measured pre-dose PEF
	PCC (range)	ICC (range)	ICC (range)
RubaTinA-asthma®^a^	0.826–0.831	0.558–0.601	0.683–0.794
PensieTinA-asthma®^b^	0.821–0.838	0.634–0.691	0.718–0.724
GraziaTinA-asthma®^c^	0.822–0.835	0.818–0.840	0.834–0.841
MezzoTinA-asthma®^d^	0.837–0.852	0.758–0.776	0.833–0.846
CadenTinA-asthma®^e^	0.773–0.791	0.741–0.769	0.780–0.825
PrimoTinA-asthma®^f^	In-clinic pre-dose PEF was not assessed	0.778–0.792	In-clinic pre-dose PEF was not assessed

Time points for measurements: ^a^Weeks 12, 24 and 48. ^b^Weeks 4, 8 and 12. ^c^Weeks 4, 8 and 12. ^d^Weeks 4, 8, 16 and 24. ^e^Weeks 12, 24, 36 and 52. ^f^Weeks 4, 8, 16, 24, 32, 40 and 48

FEV_1_ = forced expiratory volume in 1 s; ICC = intraclass correlation coefficient; PCC = Pearson correlation coefficient; PEF = peak expiratory flow

Correlation between supervised in-clinic and unsupervised home measurements was generally higher for pre-dose PEF than for pre-dose FEV_1_. For pre-dose FEV_1_, the ICCs between in-clinic responses and home-measured weekly mean responses ranged from 0.558 to 0.840 across all trials and all time points (Table 3 and Additional file 1: Table S2). For pre-dose PEF, the ICCs between in-clinic responses and home-measured weekly mean responses ranged from 0.683 to 0.846 across all trials and all time points (Table 3 and Additional file 1: Table S3). For both variables, correlations between supervised in-clinic and unsupervised home measurements were higher in the trials in adults (ICCs pre-dose FEV_1_: 0.741 to 0.840, ICCs pre-dose PEF: 0.780 to 0.846) than in the trials in adolescents (ICCs pre-dose FEV_1_: 0.558 to 0.691, ICCs pre-dose PEF: 0.683 to 0.794); see Table 3 and Additional file 1: Tables S2 and S3. For the scatter plots that visualise the correlation at the time of primary efficacy evaluation (for CadenTinA-asthma®, Week 24 was used for the analysis) across all trials, see Fig. 2.

## Discussion

The correlation analyses of pre-dose FEV_1_ and pre-dose PEF, measured under supervision in the clinic or unsupervised at home, were based on the data from 4525 patients aged 12 to 75 years who were evaluated for efficacy in the 8 Phase III parallel-group trials of the global clinical development programme with tiotropium Respimat® in asthma. Major strengths of these analyses are that the data originated from one clinical development programme, offering a high degree of consistency of trial design, and included a large number of patients, representing broad ranges of age, race, and asthma severities. However, the different trial designs and study durations mean that correlation data are not available for all time points for all studies.

Our results indicated a strong association between pre-dose FEV_1_ and pre-dose PEF when both parameters were measured under supervision in the clinic, with the PCC being greater than 0.773 across all trials and all time points. Although other studies have found only moderate correlations between PEF values and FEV_1_ [14], our results are consistent with another analysis based on data from more than 1,500 patients with asthma aged ≥15 years from two 1-year trials with montelukast, which found a mean PCC of 0.85 for the relationship between in-clinic FEV_1_ and in-clinic PEF [15]. These results support using PEF as a suitable lung function endpoint in clinical trials with asthma and a possible alternative to the more established endpoint of FEV_1_. Compared with FEV_1_, PEF has the advantage of being more broadly available to clinicians. A potential weakness of PEF, however, is that it lacks accurate reference values for many populations [1], and that reference values are specific to each brand of peak flow meter [3]. Both lung function parameters can be used to derive important information about the level of airflow obstruction initially, and in response to treatment [3].

In clinical trials, home measurements could simplify procedures and reduce the logistical burden for participating patients by decreasing the number of clinic visits required. A downside of home measurements could, however, be the dependency of the lung function values on the patient’s effort. A clear strength of ambulatory recordings of FEV_1_ or PEF is that these data provide objective and very frequent day-to-day measures of airway obstruction [1], and their weekly mean values offer robust data on patients’ lung function. When assessing the association between supervised in-clinic and unsupervised home measurements, the correlation was stronger for pre-dose PEF (ICC ≥0.683) than for pre-dose FEV_1_ (ICC ≥0.558) and for both parameters higher in adults (PEF: ICC ≥0.780, FEV_1_: ICC ≥0.741) than in adolescents (PEF: ICC ≥0.683, FEV_1_: ICC ≥0.558). This indicates that, as a lung function endpoint for self-measurement at home, PEF may be more suitable than FEV_1_. Home-measured PEF as an appropriate lung function endpoint for asthma trials is supported by the finding that longitudinal correlations between changes in asthma diary scores were stronger for average daily PEF than for weekly clinic FEV_1_ [16].

Trials in children aged < 12 years were not included in this analysis because, even with careful training, results from home spirometry in children may be less consistent [1]. However, it should be noted that home-measured PEF has been successfully used as a primary outcome measure in children previously [17].

Our results support the use of home-measured PEF in clinical asthma trials in adolescent and adult patients, potentially not only as a secondary or further outcome variable as recommended for National Institutes of Health-initiated clinical research [14], but also for consideration as a primary outcome variable. This could improve patients’ acceptance and willingness to participate in clinical trials by facilitating procedures and reducing the logistical burden for them by relocating scheduled assessments from the clinic to their home. It also supports respiratory clinical trials that are more geared towards patient involvement or follow a real-world pragmatic approach, with the potential opportunity to recruit patients who would not have been able to participate otherwise. This finding would have to be implemented in regulatory guidelines.

## Conclusions

In conclusion, this post hoc analysis supports pre-dose PEF, measured under supervision in the clinic or unsupervised at home, as an alternative primary lung function endpoint for trials in adolescent and adult patients with asthma.

## Additional file

Additional file 1: **Table S1**. Correlation analysis results (Pearson correlation coefficient) at different time points between pre-dose FEV_1_ and pre-dose PEF, both measured under supervision in the clinic. Phase III trials with tiotropium Respimat® in patients aged 12–75 years with persistent asthma – all patients evaluated for efficacy. **Table S2**. Correlation analysis results (intraclass correlation coefficient) at different time points between supervised measurement in the clinic and unsupervised measurement at home for pre-dose FEV_1_. Phase III trials with tiotropium Respimat® in patients aged 12–75 years with persistent asthma – all patients evaluated for efficacy. **Table S3**. Correlation analysis results (intraclass correlation coefficient) at different time points between supervised measurement in the clinic and unsupervised measurement at home for pre-dose PEF. Phase III trials with tiotropium Respimat® in patients aged 12–75 years with persistent asthma – all patients evaluated for efficacy. (DOCX 20 kb)

## Data Availability

The data sets used and/or analysed during the current study are available from the corresponding author on reasonable request.

## Abbreviations

ATS: American Thoracic Society
ERS: European Respiratory Society
ERT: eResearch Technology
FEV_1_: forced expiratory volume in 1 s
ICC: intraclass correlation coefficient
ICS: inhaled corticosteroid
PCC: Pearson correlation coefficient
PEF: peak expiratory flow
