# Supplementary material for: Peak expiratory flow as an endpoint for clinical trials in asthma: a comparison with FEV1
Source: Respir Res. 2019 Jul 18;20:159. doi: 10.1186/s12931-019-1119-6 (PMC6637596; doi:10.1186/s12931-019-1119-6)
Supplement: Supplementary file 1 — Table S1. Correlation analysis results (Pearson correlation coefficient) at different time points between pre-dose FEV1 and pre-dose PEF, both measured under supervision in the clinic. Phase III trials with tiotropium Respimat® in patients aged 12–75 years with persistent asthma – all patients evaluated for efficacy. Table S2. Correlation analysis results (intraclass correlation coefficient) at different time points between supervised measurement in the clinic and unsupervised measurement at home for pre-dose FEV1. Phase III trials with tiotropium Respimat® in patients aged 12–75 years with persistent asthma – all patients evaluated for efficacy. Table S3. Correlation analysis results (intraclass correlation coefficient) at different time points between supervised measurement in the clinic and unsupervised measurement at home for pre-dose PEF. Phase III trials with tiotropium Respimat® in patients aged 12–75 years with persistent asthma – all patients evaluated for efficacy. (DOCX 20 kb) [file 12931_2019_1119_MOESM1_ESM.docx]

**Supplementary material**

Table S1. Correlation analysis results (Pearson correlation coefficient) at different time points between pre-dose FEV_1_ and pre-dose PEF, both measured under supervision in the clinic. Phase III trials with tiotropium Respimat^®^ in patients aged 12–75 years with persistent asthma – all patients evaluated for efficacy.

| **Time point, week** | **Adolescent studies** | | **Adult studies*** | | |
| --- | --- | --- | --- | --- | --- |
|  | **RubaTinA-asthma®** | **PensieTinA-asthma®** | **GraziaTinA-asthma®** | **MezzoTinA-asthma®** | **CadenTinA-asthma®** |
| 4 |  | 0.821 | 0.822 | 0.837 |  |
| 8 |  | 0.838 | 0.822 | 0.842 |  |
| 12 | 0.826 | 0.827 | 0.835 |  | 0.776 |
| 16 |  |  |  | 0.837 |  |
| 24 | 0.828 |  |  | 0.852 | 0.791 |
| 36 |  |  |  |  | 0.773 |
| 48 | 0.831 |  |  |  |  |
| 52 |  |  |  |  | 0.783 |

*PEF was not measured at clinic visits in the PrimoTinA-asthma® study.

FEV_1_ = forced expiratory volume in 1 second; PEF = peak expiratory flow.

Table S2. Correlation analysis results (intraclass correlation coefficient) at different time points between supervised measurement in the clinic and unsupervised measurement at home for pre-dose FEV_1_. Phase III trials with tiotropium Respimat^®^ in patients aged 12–75 years with persistent asthma – all patients evaluated for efficacy.

| **Time point, week** | **Adolescent studies** | | **Adult studies** | | | |
| --- | --- | --- | --- | --- | --- | --- |
|  | **RubaTinA-asthma®** | **PensieTinA-asthma®** | **GraziaTinA-asthma®** | **MezzoTinA-asthma®** | **CadenTinA-asthma®** | **PrimoTinA-asthma®** |
| 4 |  | 0.634 | 0.825 | 0.773 |  | 0.790 |
| 8 |  | 0.691 | 0.840 | 0.776 |  | 0.792 |
| 12 | 0.601 | 0.681 | 0.818 |  | 0.769 |  |
| 16 |  |  |  | 0.758 |  | 0.778 |
| 24 | 0.575 |  |  | 0.775 | 0.741 | 0.792 |
| 32 |  |  |  |  |  | 0.790 |
| 36 |  |  |  |  | 0.765 |  |
| 40 |  |  |  |  |  | 0.779 |
| 48 | 0.558 |  |  |  |  | 0.785 |
| 52 |  |  |  |  | 0.766 |  |

FEV_1_ = forced expiratory volume in 1 second.

Table S3. Correlation analysis results (intraclass correlation coefficient) at different time points between supervised measurement in the clinic and unsupervised measurement at home for pre-dose PEF. Phase III trials with tiotropium Respimat^®^ in patients aged 12–75 years with persistent asthma – all patients evaluated for efficacy.

| **Time point, week** | **Adolescent studies** | | **Adult studies*** | | |
| --- | --- | --- | --- | --- | --- |
|  | **RubaTinA-asthma®** | **PensieTinA-asthma®** | **GraziaTinA-asthma®** | **MezzoTinA-asthma®** | **CadenTinA-asthma®** |
| 4 |  | 0.718 | 0.841 | 0.843 |  |
| 8 |  | 0.720 | 0.834 | 0.846 |  |
| 12 | 0.794 | 0.724 | 0.839 |  | 0.825 |
| 16 |  |  |  | 0.835 |  |
| 24 | 0.740 |  |  | 0.833 | 0.806 |
| 36 |  |  |  |  | 0.792 |
| 48 | 0.683 |  |  |  |  |
| 52 |  |  |  |  | 0.780 |

*PEF was not measured at clinic visits in the PrimoTinA-asthma® study.

PEF = peak expiratory flow.
